# Complete genome sequence of *Ilumatobacter coccineum* YM16-304^T^

**DOI:** 10.4056/sigs.4007734

**Published:** 2013-07-30

**Authors:** Shun Fujinami, Hiromi Takarada, Hiroaki Kasai, Mitsuo Sekine, Seiha Omata, Takeshi Harada, Rieko Fukai, Akira Hosoyama, Hiroshi Horikawa, Yumiko Kato, Hidekazu Nakazawa, Nobuyuki Fujita

**Affiliations:** 1 Biological Resource Center, National Institute of Technology and Evaluation, Shibuya, Tokyo, Japan; 2 Bio-Nano Electronics Research Centre, Toyo University, 2100 Kujirai, Kawagoe Saitama, Japan; 3 Marine Biosciences Kamaishi Research Laboratory, Kitasato University, Ofunato, Iwate, Japan

**Keywords:** aerobic, mesophilic, marine sponge

## Abstract

*Ilumatobacter coccineum* YM16-304^T^ (=NBRC 103263^T^) is a novel marine actinobacterium isolated from a sand sample collected at a beach in Shimane Prefecture, Japan. Strain YM16-304^T^ is the type strain of the species. Phylogenetically, strain YM16-304^T^ is close to *Ilumatobacter nonamiense* YM16-303^T^ (=NBRC 109120^T^), *Ilumatobacter fluminis* YM22-133^T^ and some uncultured bacteria including putative marine sponge symbionts. Whole genome sequence of these species has not been reported. Here we report the complete genome sequence of strain YM16-304^T^. The 4,830,181 bp chromosome was predicted to encode a total of 4,291 protein-coding genes.

## Introduction

**Figure 1:**
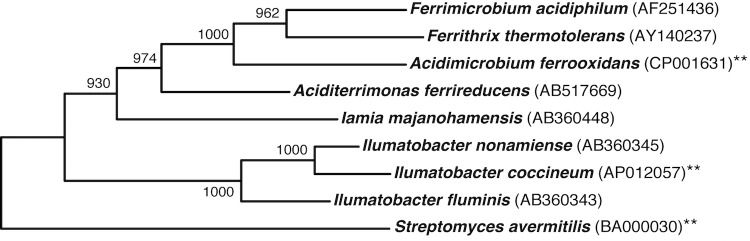
Phylogenetic tree highlighting the position of *I. coccineum* strain YM16-304^T^ relative to other representative type strains. The tree was constructed by the neighbor-joining method [3] based on a 1,326 bp alignment of 16S rRNA gene sequences. Corresponding INSDC accession numbers are shown in parentheses. Numbers at nodes indicate support values obtained from 1,000 bootstrap replications. Species for which complete genome sequences are available are labeled with two asterisks.

Strain YM16-304^T^ (=NBRC 103263^T^) is the type strain of *Ilumatobacter coccineum* Matsumoto *et al*. 2013 [1]. *I. coccineum* YM16-304^T^ and *Ilumatobacter nonamiense* YM16-303^T^ (=NBRC 109120^T^) were isolated from a sand sample collected at Nonami Beach in Shimane Prefecture in Japan, and represent the second and the third species of the genus *Ilumatobacter* [2]. Phylogenetic analysis showed that genus *Ilumatobacter* branches near the presumed root of the class *Actinobacteria* (Figure 1), and thus may represent a new taxon outside the known family *Acidimicrobiaceae*, although the family accommodating this genus has not been decided yet [1,2]. *Iamia majanohamensis* is also located outside the family *Acidimicrobiaceae*, and is the sole genus and species in family *Iamiaceae* [4]. Among the organisms for which whole genome sequences have been reported, the most closely related to YM16-304^T^ is *Acidimicrobium ferrooxidans* DSM 10331 [5], which is phylogenetically distant from *I. coccineum* with 16S rRNA gene sequence similarity of 86%. No complete or draft genome information is currently available for the genera *Ilumatobacter* and *Iamia*. The taxon contains a number of uncultured bacteria including putative marine sponge symbionts, and the complete genome sequence of strain YM16-304^T^ would provide a basis of technological developments for the isolation and better understanding of related uncultured actinobacteria.

## Classification and features

**Figure 2:**
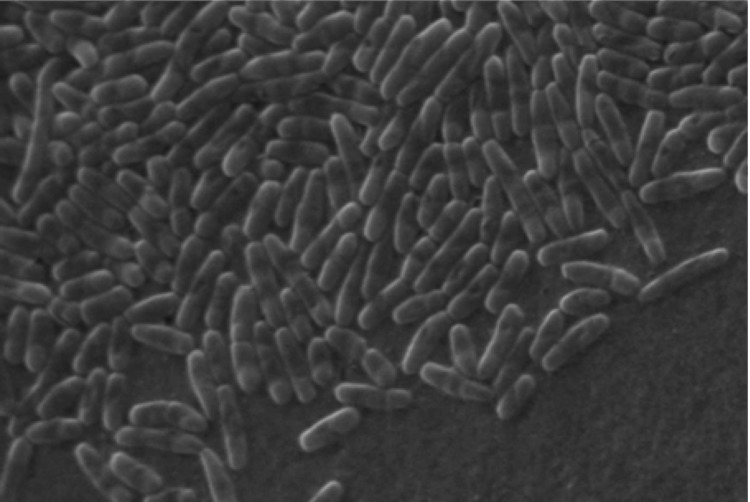
Scanning electron micrograph of *I. coccineum* strain YM16-304^T^

Strain YM16-304^T^ is a mesophilic, neutrophilic, aerobic bacterium with features as summarized in Table 1. Growth occurs at 12 – 36 °C and at pH 7-8. Cells are rods and non-motile [Figure 2]. Gram staining was positive. Electron microscope observation demonstrated no flagella and pili formation [1]. In agreement with this observation, the genome encodes no gene necessary for flagella, chemotaxis and pili.

**Table 1:** Classification and general features of *I. coccineum* strain YM16-304^T^

**MIGS ID**	**Property**	**Term**	**Evidence code**^a^
	Current classification	Domain *Bacteria*	TAS [6]
		Phylum *Actinobacteria*	TAS [7]
		Class *Actinobacteria*	TAS [8]
		Subclass *Acidimicrobidae*	TAS [8,9]
		Order *Acidimicrobiales*	TAS [8,9]
		Family unclassified	TAS [1]
		Genus *Ilumatobacter*	TAS [2,10]
		Species *Ilumatobacter coccineum*	TAS [1]
		Type strain YM16-304	TAS [7]
	Gram stain	positive	TAS [1]
	Cell shape	rods	TAS [1]
	Motility	non-motile	TAS [1]
	Sporulation	not reported	
	Temperature range	12-36°C	TAS [1]
	Optimum temperature	16-24°C	TAS [1]
	Carbon source	Peptone, yeast extract	TAS [1]
	Energy source	heterotrophic	TAS [1]
MIGS-6	Habitat	beach sand	TAS [1]
MIGS-6.3	Salinity	salt tolerant	TAS [1]
MIGS-22	Oxygen	aerobic	TAS [1]
MIGS-15	Biotic relationship	free-living	NAS
MIGS-14	Pathogenicity	none	NAS
MIGS-4	Geographic location	Nonami Beach, Shimane Pref., Japan	TAS [1]
MIGS-5	Sample collection time	March 2005	TAS [1]
MIGS-4.1	Latitude	N 35º34’42”	TAS [1]
MIGS-4.2	Longitude	E 133º05’48”	TAS [1]
MIGS-4.3	Depth	No record	
MIGS-4.4	Altitude	NA	

a) Evidence codes - TAS: Traceable Author Statement (i.e., a direct report exists in the literature); NAS: Non-traceable Author Statement (i.e., not directly observed for the living, isolated sample, but based on a generally accepted property for the species, or anecdotal evidence). These evidence codes are from the Gene Ontology project [11].

Strain YM16-304^T^ grows poorly even in artificial seawater medium supplemented with 0.5% peptone and 0.1% yeast extract under optimum growth conditions [1]. From the genome sequence, strain YM16-304^T^ seems to possess either deficient or unusual pathways for the synthesis of some amino acids and other essential cellular components as outlined in the later section (Primary metabolism).

## Genome sequencing information

### Genome project history

*I. coccineum* YM16-304^T^ was selected for sequencing because of its isolated phylogenetic position and characteristics which distinguish this strain from other described actinobacterial species. Table 2 presents the project information and its association with MIGS version 2.0 compliance [15].

**Table 2:** Project information

**MIGS ID**	**Property**	**Term**
MIGS-31	Finishing quality	finished
MIGS-28	Libraries used	two plasmid libraries with average insert sizes of 1.5 kb and 6.0 kb and a fosmid library with average insert size of 38 kb
MIGS-29	Sequencing platforms	ABI 3730xl
MIGS-31.2	Fold coverage	8.1 ×
MIGS-30	Assemblers	Phrap [12,13]
MIGS-32	Gene calling method	Glimmer3 [14]
	INSDC ID	AP012057
	Genbank Date of Release	March 16, 2013
	NCBI project ID	PRJDA63297
	GOLD ID	Gi02040 (to be updated)
MIGS-13	Source material identifier	NBRC 103263
	Project relevance	biotechnology, systematics

### Growth conditions and DNA isolation

*I. coccineum* YM16-304^T^ cells were grown in a 20 L volume at 27°C in Difco^TM^ Marine broth 2216 (Beckton Dickinson). DNA was isolated from 0.5 g of wet cells by manual extraction after lysis with lysozyme and SDS.

### Genome sequencing and assembly

The genome of *I. coccineum* YM16-304^T^ was sequenced using the conventional whole-genome shotgun sequencing method. Plasmid libraries with average insert sizes of 1.5 kb and 6.0 kb were generated in pTS1 (Nippon Gene) and pUC118 (TaKaRa) vectors, respectively, while a fosmid library with average insert size of 38 kb was constructed in pCC1FOS (EPICENTRE) as described previously [16]. A total of 26,592 clones (18,432, 5,376 and 2,784 clones from libraries with 1.5 kb, 6.0 kb and 38 kb inserts, respectively) were subjected to sequencing from both ends of the inserts on a ABI 3730xl DNA Analyzer (Applied Biosystems). Sequence reads were trimmed at a threshold of 20 in Phred score and assembled by using Phrap and CONSED assembly tools [12,13]. Gaps between contigs were closed by sequencing PCR products which bridge two neighboring contigs. Finally, each base of the genome was ensured to be sequenced from multiple clones either from both directions with Phrap quality score ≥ 70 or from one direction with Phrap quality score ≥40.

### Genome annotation

The complete sequence of the chromosome was analyzed using Glimmer3 [14] for predicting protein-coding genes, tRNAscan-SE [17] and ARAGORN [18] for tRNA genes, and RNAmmer [19] for rRNA genes. The functions of predicted protein-coding genes were assigned manually, using the in-house genome annotation system OCSS (unpublished), in comparison with Uniprot [20], Interpro [21], HAMAP [22] and KEGG [23] databases.

## Genome properties

**Figure 3:**
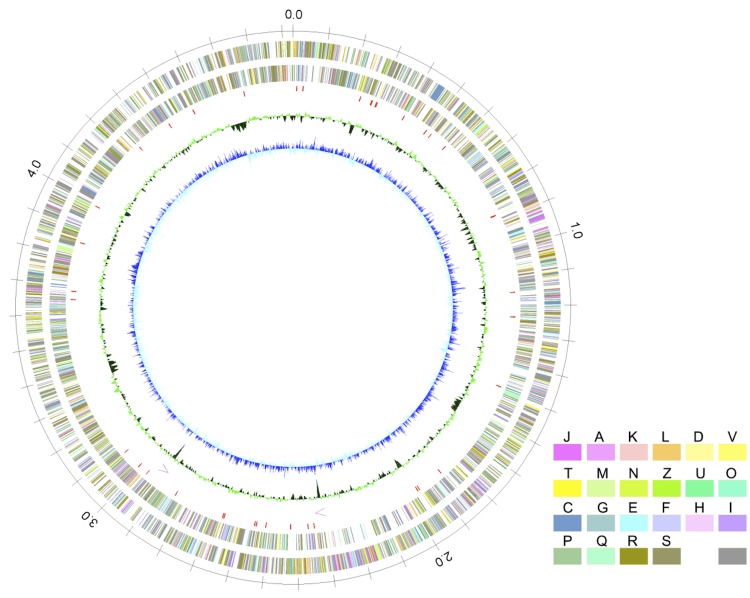
Circular representation of the *I. coccineum* YM16-304^T^ chromosome From outside to the center: circles 1 and 2, predicted protein coding genes on the forward and reverse strands, respectively; circle 3, tRNA genes; circle 4, rRNA operons; circle 5, G+C content; circle 6, GC skew. Predicted protein coding genes are colored according to their assigned COG functional categories (see Table 4).

The genome of *I. coccineum* YM16-304^T^ consisted of a circular chromosome of 4,830,181 bp (Figure 3). The chromosome was predicted to contain 4,291 protein-coding genes, 46 tRNA genes, two copies of rRNA operons. Protein functions were manually assigned based on UniProt and InterPro searches, and specific functions were predicted for 1,824 genes (42.5% of the protein-coding genes). Among the remaining predicted proteins, 520 (12.1%) were assigned to proteins belonging to specific protein families, 1,535 (35.8%) were assigned to hypothetical proteins (showing sequence similarity to published proteins without known function), and 409 (9.5%) were assigned to hypothetical proteins (prediction only) (lacking sequence similarity to published proteins). Average G+C content was 67.29%. The properties and the statistics of the genome are summarized in Tables 3-4.

**Table 3:** Nucleotide content and gene count levels of the genome

**Attribute**	Value	% of total^a^
Genome size (bp)	4,830,181	100.00%
DNA Coding region (bp)	4,490,739	92.97%
DNA G+C content (bp)	3,250,347	67.29%
Number of replicons	1	
Extrachromosomal elements	0	
Total genes	4,346	
RNA genes	55	
rRNA operons	2	
Protein-coding genes	4,291	100.00%
Pseudo genes	0	0.00%
Genes with function prediction	2,342	54.58%
Genes in paralog clusters	1,140	26.57%
Genes assigned to COGs	3,186	74.25%
Genes assigned Pfam domains	3,053	71.15%
Genes with signal peptides	401	9.35%
Genes with transmembrane helices	876	20.41%
Paralogous groups	300	% of totala

a) The total is based on either the size of the genome in base pairs or the total number of protein coding genes in the annotated genome.

**Table 4:** Number of genes associated with the 25 general COG functional categories

**Code**	**Value**	**%age**^a^	**Description**
J	165	3.85	Translation
A	3	0.07	RNA processing and modification
K	283	6.60	Transcription
L	129	3.01	Replication, recombination and repair
B	3	0.07	Chromatin structure and dynamics
D	47	1.10	Cell cycle control, mitosis and meiosis
Y	0	0.00	Nuclear structure
V	57	1.33	Defense mechanisms
T	162	3.78	Signal transduction mechanisms
M	176	4.10	Cell wall/membrane biogenesis
N	9	0.21	Cell motility
Z	8	0.19	Cytoskeleton
W	0	0.00	Extracellular structures
U	39	0.91	Intracellular trafficking and secretion
O	104	2.42	Posttranslational modification, protein turnover, chaperones
C	271	6.32	Energy production and conversion
G	203	4.73	Carbohydrate transport and metabolism
E	295	6.87	Amino acid transport and metabolism
F	80	1.86	Nucleotide transport and metabolism
H	119	2.77	Coenzyme transport and metabolism
I	208	4.85	Lipid transport and metabolism
P	205	4.78	Inorganic ion transport and metabolism
Q	183	4.26	Secondary metabolites biosynthesis, transport and catabolism
R	613	14.29	General function prediction only
S	284	6.62	Function unknown
-	1105	25.75	Not in COGs

a) The total is based on the total number of protein coding genes in the annotated genome.

### Primary metabolism

Strain YM16-304^T^ lacks the *dapE* gene for succinyl-diaminopimelate desuccinylase (EC:3.5.1.18) in the biosynthesis pathway of lysine and diaminopimelic acids (DAPs). Instead, two candidate genes (YM304_26990 and YM304_19190) for LL-DAP aminotransferase (EC:2.6.1.83, *dapL*), that constitutes an alternative DAP-lysine biosynthesis pathway (DAP aminotransferase pathway [24,25]), were identified. The *dapL* gene is found in discrete lineages of *Bacteria* and *Archaea*, and is known to complement *Escherichia coli dapD* and *dapE* mutants, although purified proteins favor the reverse reaction rather than the synthesis of LL-DAP [25].

Among the genes of serine biosynthesis pathway, the *serB* gene for phosphoserine phosphatase (EC:3.1.3.3) was not identified by similarity searches. On the other hand, the *thrH* gene for phosphoserine / homoserine phosphotransferase [26] (EC:3.1.3.3, 2.7.1.39) was identified (YM304_28950). The possibility of using *thrH* gene product for serine biosynthesis instead of *serB* gene product was suggested.

Strain YM16-304^T^seems to possess an alternative form of histidine biosynthesis pathway in which *hisB* gene for the synthesis of L-histidinol was replaced with the *hisN* gene (YM304_12240) as typically found in *Corynebacterium glutamicum* ATCC13032 [27] and other actinomycetes. However, the *hisE* gene for phosphoribosyl-ATP pyrophosphohydrolase (EC:3.6.1.31), which is responsible for the second step in histidine biosynthesis pathway, was not identified by similarity searches.

Metabolic reconstruction based on the annotation suggested that strain YM16-304^T^ possesses the enzymes required for the biosynthesis of saturated fatty acids, unsaturated fatty acids, branched-chain fatty acids and carotenoids. The putative carotenoid biosynthesis pathway comprises *crtE* (YM304_37400), *crtB* (YM304_37420), *crtI* (YM304_37410) and *crtLm* (YM304_23780) gene homologs, which most probably synthesizes γ-carotene from isopentenyl pyrophosphate derived from non-mevalonate pathway [28-30]. Strain YM16-304^T^ also possesses genes homologous to *crtO* (YM304_25370) and *crtZ* (YM304_38780), which were suggested to be involved in the synthesis of ketolated carotenoid such as canthaxanthin and astaxanthin [30]. Actual products of this pathway need to be experimentally verified.

The annotation also suggests that strain YM16-304^T^ possesses the enzymes required for the biosynthesis of menaquinone (vitamin K), vitamin B_6_, nicotinate and nicotinamide, pantothenate and CoA, lipoic acid, protoheme, mycothiol and coenzyme F_420_, while biosynthetic pathways for folate, thiamine, riboflavin, biotin and adenosylcobalamin (coenzyme B_12_) are either missing or incomplete.

### Secondary metabolism

The phylogenetic analysis based on 16S rRNA gene sequences showed that three species in the genus *Ilumatobacter* were closely related to some uncultured actinobacteria including marine sponge symbionts [31]. Marine sponges are noted as a rich source of biologically active secondary metabolites, true producers of such compound being suspected to be symbiotic bacteria [32-34]. However, only a small percentage of these symbiotic microorganisms are culturable [35,36], and genes involved in the synthesis of bioactive compounds such as polyketide synthases have often been isolated by metagenomic approaches [37,38].

The strain YM16-304^T^ genome seemed to encode only a limited number of secondary metabolic enzymes, i.e., two type I polyketide synthases (PKS). The genome does not contain genes for type II and type III PKS nor a gene for nonribosomal peptide synthetase.

The type I PKS genes of the strain YM16-304^T^ (YM304_13420, YM304_13410), together with the adjacent *pfaD* homolog (YM304_13430), most probably encode omega-3 polyunsaturated fatty acid (PUFA) synthase gene cluster. In some *Gammaproteobacteria* from marine sources such as *Photobacterium profundum* strain SS9, omega-3 polyunsaturated fatty acids such as eicosapentaenoic acid (20:5n-3; EPA) and docosahexaenoic acid (22:6n-3; DHA) are known to be synthesized by a PKS system consisting of *pfaA, pfaB, pfaC* and *pfaD* genes [39-41]. The domain organization of YM304_13420 was identical to that of the *pfaA* gene of *P. profundum* SS9. The N-terminal ketosynthase domain and the C-terminal dehydratase domains of YM304_13410 were similar to those of the *pfaC* gene of *P. profundum*, while the internal acyltransferase domain of YM304_13410 was moderately similar to that of the *pfaB* gene of *P. profundum*, representing a presumed chimeric form of PKS. As PfaB is the key enzyme determining the final product in EPA or DHA biosynthesis [42], the actual product of this PKS system may need to be clarified experimentally. Some PUFA-producing bacteria such as *Moritella marina* MP-1 [39,43] were reported to require an additional gene, *pfaE*, encoding a phosphopantheteinyl transferase. However, the *pfaE* gene was not identified in strain YM16-304^T^. Other classes of phosphopantheteinyl transferase (e.g. YM304_08850) may substitute the function of PfaE, similar to the case suggested in *P. profundum* SS9 [44].

### Cell surface

Strain YM16-304 seemed to possess 13 ORFs containing LPXTG motif (InterPro ID: IPR001899), the presumed sorting signal of cell surface proteins in Gram-positive bacteria [45]. It was reported that several cell surface proteins containing LPXTG motif act as an adhesion factor known as microbial surface components recognizing adhesive matrix molecules (MSCRAMMs) [46]. The genome of strain YM16-304 contained extracellular polysaccharide gene cluster (YM304_29910- YM304_30490), including gene cluster for the synthesis of sialic acids (YM304_30300- YM304_30320), which are also crucial for cell adhesion [47]. These extracellular components might serve for the bacterium to adhere to host tissues such as marine sponges.

Many marine bacteria use the Na^+^ cycle and require Na^+^ for their growth [48]. In these bacteria, Na^+^ is often used in the respiratory chain, ATP synthase, flagellar rotation and solute uptake instead of H^+^ [49]. Some bacteria can use both Na^+^ and H^+^ to expand the range of environments in which the bacteria can grow [50]. Strain YM16-304 was isolated from a sand sample collected at a beach and grows optimally in marine broth media, suggesting its marine origin. However, the gene products for the respiratory chain and ATP synthase were predicted to be of the H^+^-dependent type by similarity search. The Na^+^-dependent amino acid symporters were also not identified, nor was the H^+^-dependent symporters.
